# Fashion vs. Function in Cultural Evolution: The Case of Dog Breed Popularity

**DOI:** 10.1371/journal.pone.0074770

**Published:** 2013-09-11

**Authors:** Stefano Ghirlanda, Alberto Acerbi, Harold Herzog, James A. Serpell

**Affiliations:** 1 Department of Psychology, Brooklyn College, Brooklyn, New York, United States of America; 2 Centre for the Study of Cultural Evolution, University of Stockholm, Stockholm, Sweden; 3 Department of Archaeology and Anthropology, University of Bristol, Bristol, United Kingdom; 4 Department of Psychology, Western Carolina University, Cullowhee, North Carolina, United States of America; 5 School of Veterinary Medicine, University of Pennsylvania, Philadelphia, Pennsylvania, United States of America; Bristol University, United Kingdom

## Abstract

We investigate the relationship between characteristics of dog breeds and their popularity between years 1926 and 2005. We consider breed health, longevity, and behavioral qualities such as aggressiveness, trainability, and fearfulness. We show that a breed's overall popularity, fluctuations in popularity, and rates of increase and decrease around popularity peaks show typically no correlation with these breed characteristics. One exception is the finding that more popular breeds tend to suffer from more inherited disorders. Our results support the hypothesis that dog breed popularity has been primarily determined by fashion rather than function.

## Introduction

The popularity of dog breeds shows, over time, the kind of large and apparently whimsical fluctuations that are usually considered the hallmark of fashions and fads [1], [2]. Registrations of Irish setter puppies with the American Kennel Club, for example, climbed from about 2,500 in 1961 to over 60,000 in 1974, only to drop to about 3,000 by 1986. Many other breeds exhibit similar fluctuations [2]–[4]. Theoretical models of cultural dynamics have viewed such fluctuations as the result of either chance [1], [5], [6] or of shifts in population preferences that occur when many individuals imitate a few influential cultural models [7]. Both explanations deny that the intrinsic characteristics of breeds which would presumably make for good pets, such as their temperament or longevity, play any role in determining which breeds are popular at any given time. This claim may appear paradoxical given the important place that dogs hold in the life of many, and is as yet untested.

Here we investigate the relationships between breed characteristics and breed popularity by collating popularity data from the American Kennel Club's dog registry, behavior data from the Canine Behavioral Assessment and Research Questionnaire (C-BARQ), and longevity and health data from several sources (see Methods). We find no indication that behavior, health, or longevity have been important in determining breed popularity. The data show, rather, that the most popular breeds have significant health problems, and possibly more behavioral problems. We conclude by discussing the significance of these results for animal welfare and a broader understanding of cultural dynamics.

## Methods

### Popularity Data

The American Kennel Club (AKC) encourages dog owners to register their dogs in the AKC database. The AKC has provided us with registration data from 1926 to 2005, totaling over 50 million dogs from ∼150 recognized breeds (see [1], [8] for further details, data available at http://dx.doi.org/10.6084/m9.figshare.715895). These data can be represented in matrix form, with 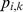 the number of dogs of breed 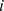 registered with the AKC in year 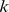 (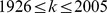). From these data we have derived four measures of breed popularity:

- Total popularity, defined as the total number of registrations for each breed in 1926–2005,

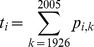

- Volatility, defined as the average absolute change in registrations from one year to the next:

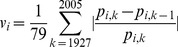
where 79 is the number of registration changes that can be computed from available data.

- Rates of increase in registrations before peaks in popularity.
- Rates of decrease in registration after peaks in popularity.

The latter two measures are defined according to [7], [9]. Namely, a putative peak is identified as the maximum of registrations. The beginning (end) of the peak is identified as the first time before (after) the peak year that registrations are at 10% or less of peak value. If the beginning and end of the peak cannot be located thus, the breed is deemed as having had no peak. If 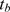, 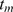 are the years marking the beginning and the of a peak, rates of popularity increase are defined as.
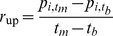



Rates of popularity decrease after a peak, 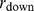 are defined similarly. Visual inspection of the data reveals that some breeds have had two or three peaks, but here we consider only the highest one to avoid possible ambiguity in identifying these secondary peaks.

### Behavioral Data

Data about breed behavior and temperament where obtained from the C-BARQ database [10]–[12]. C-BARQ evaluates dog behavior and temperament on 14 scales, summarized in Table 1, and has high validity and reliability [10], [12], [13]. At the time of data analysis, the database contained information from 12,059 dogs, of which 9,824 were of known breed (data available at http://dx.doi.org/10.6084/m9.figshare.715896). We eliminated breeds with fewer than 20 individuals, leaving 9,046 dogs from 92 breeds. AKC data were available for 80 of these breeds, corresponding to 8,645 dogs in C-BARQ. We obtained breed-typical scores from these data by averaging C-BARQ scores for all dogs of the same breed.

**Table 1:** Brief description of C-BARQ behavior and temperament scales (see [10], [12] for details).

C-BARQ variable	Example behaviors
Trainability (trainability)	Dog returns when called, obeys “sit” and “stay” commands, quick to learn new tricks, not easily distracted
Stranger-directed aggression (strangeraggr)	Dog acts aggressively when approached by unfamiliar person, when unfamiliar person approaches owner or family member outdoors or at home
Owner-directed aggression (owneraggr)	Dog acts aggressively when members of the household challenge, manhandle, stare at, step over, or approach when in possession of food or toys
Dog rivalry (dogrivalry)	Dog acts aggressively toward (familiar) dogs in the household when resting at a favorite place, eating, playing with favorite toy
Stranger-directed fear (strangerfear)	Dogs acts fearful or anxious when an unfamiliar person approaches outside the home, visits the home, tries to touch or pet the dog
Nonsocial fear (nonsocialfear)	Dogs acts fearful or anxious in response to loud noises, heavy traffic, unfamiliar objects, thunderstorms, unfamiliar situations
Dog-directed aggression (dogaggr)	Dog acts aggressively when an unfamiliar dog approaches directly at the home or when being walked
Dog-directed fear (dogfear)	Dog acts anxious or fearful when an unfamiliar dog approaches directly, visits the home, barks or growls at the dog
Touch sensitivity (touchsens)	Dog acts anxious or fearful when examined or treated by a veterinarian, when groomed or bathed by a household member
Separation-related behavior (sepprobs)	When left alone or about to be left alone dog shivers, trembles, salivates excessively, is agitated, barks or howls, chews or scratches doors, curtains, floor
Excitability (excitability)	Dog acts excited when member of household returns after brief absence or plays with dog, when doorbell rings, just before being taken for a walk or car trip
Attachment/attention seeking (attachatten)	Dogs tends to follow a member of household from room to room, tends to sit close, becomes agitated when a member of household shows affection for another person, dog, or other animal
Chasing (chasing)	Dog shows predatory behavior toward cats, squirrels or other animals, would chase cats, birds, squirrels, rabbits if given the opportunity
Energy level (energy)	Dog is playful, puppyish, boisterous, active, energetic

**Figure 1:**
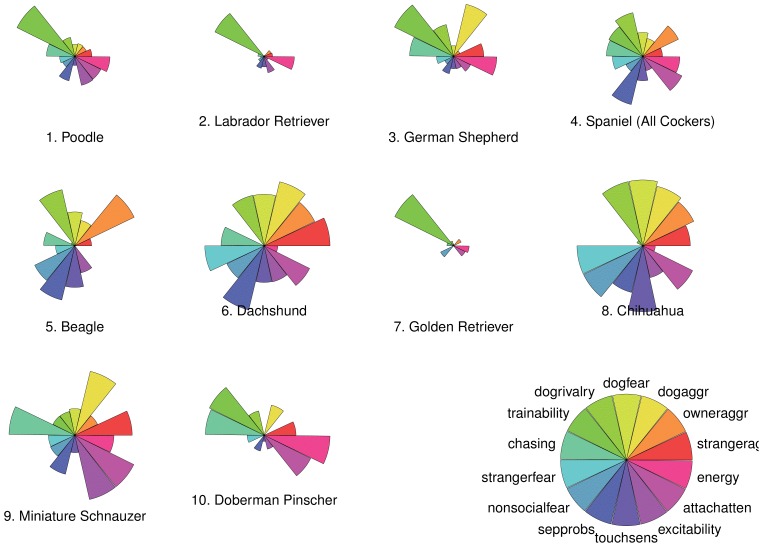
Behavior and temperament measures for the 10 most popular dog breeds. Behavior and temperament measures for the 10 most popular dog breeds in the AKC database, years 1926–2005. Measures are defined in Table 1.

**Figure 2:**
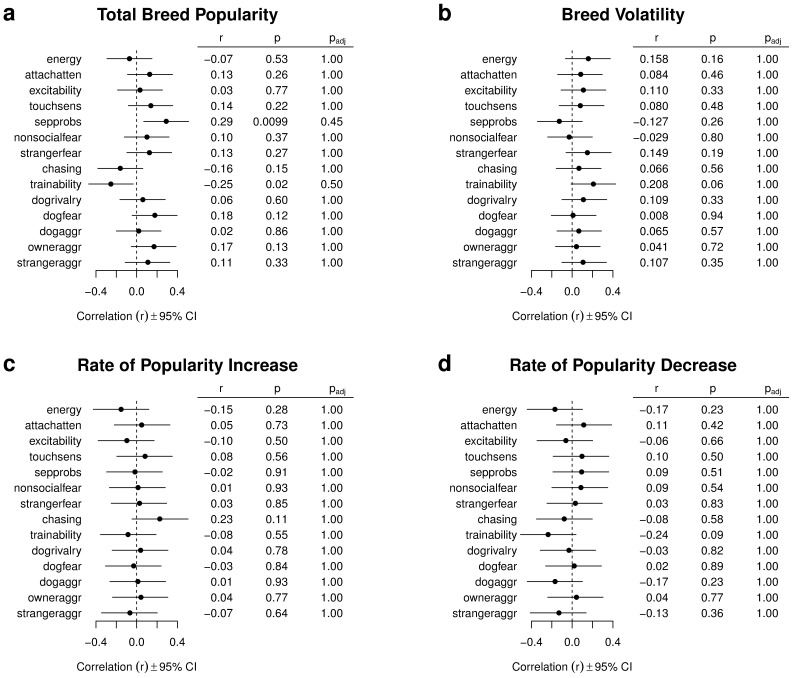
Correlations between measures of breed popularity and behavior variables from the C-BARQ questionnaire. Each panel displays Pearson's correlations between one measure of breed popularity (total number of dogs, breed volatility, rates of increase and decrease around popularity peaks) and the 14 behavioral variables assessed by the C-BARQ questionnaire (Table 1). The correlations are displayed both graphically and numerically in the first table column. Confidence intervals (displayed graphically) and 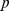 values (reported in the second table column) are bootstrapped owing to non-normality; 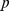 values adjusted to control the false discovery rate [19] are reported in the third table column. The sample size for all correlations is 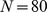.

The short codes used in Figures 1 and 2 are given in parenthesis in the first column.

The analysis below assumes that the breed-typical behaviors derived from the C-BARQ database have not changed greatly over the 80 years spanned by AKC data. We cannot prove this assumption, but we have run the analysis reported below considering popularity data from the decade 1996–2005 only, and found no difference in results.

### Longevity Data

Median breed longevity was obtained from published reports based on veterinary hospital records [14], and from aggregations of single-breed and multi-breed surveys [15]. When both sources are available, veterinary hospital records yield always lower estimates than surveys (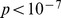, two-tailed Wilcoxon signed rank test), likely because veterinary hospitals primarily see ill dogs. Longevity data from either source were available for 67 breeds.

### Health Data

Asher et al. [16] and Summers et al. [17] collated evidence from online databases and published sources seeking to characterize inherited disorders affecting the 50 breeds that were most popular in the U.K. around 2007. These authors categorized disorders as those that resulted from selection to adhere to breed standards (or that were aggravated by such selection), and those that, given current knowledge, appear unrelated to breed standards. Our aim is to investigate the effect of breed health on popularity and therefore we consider the total number of disorders affecting each breed. The results reported below are based on correlating the total number of disorders, provided in Table 1 of Summers et al.[17], with U.K. breed popularity data from the same source, and with U.S. popularity data from the AKC. Forty-three breeds considered in [17] are also present in AKC data. (The breed standards of the AKC and the U.K. Kennel Club only have minor differences.).

### Statistical Methods

We used Pearson's correlations to assess the relationships between measures of breed popularity and the breed's behavioral characteristics, longevity, and number of inherited disorders. We estimated confidence intervals and significance of correlations using bootstrapped permutation tests as most variables are distributed non-normally (we used 50,000 permutations of the original data for each test). We used ANOVAs to quantify breed variability in behavioral characteristics and to estimate the amount of variance in popularity accounted for by number of inherited disorders. Data analysis was performed with R version 3.0.0 [18]. Specifically, we used the boot package for bootstrap calculations, and the p.adjust function to adjust 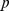 values for multiple comparisons (see below).

## Results

### Breed Variability in Behavior, Longevity and Health

A necessary condition for a given characteristic to influence breed popularity is that breeds differ appreciably in that characteristic. We performed ANOVAs with each of the C-BARQ measures in Table 1 as the dependent variable, and breed as independent variables. The effect of breed was significant for all measures, with 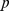-values never exceeding 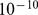. Figure 1 exemplifies such variation for the 10 most popular breeds in the AKC database. There was also substantial variation across breeds in the number of inherited disorders (median 32.5, range 10–77) and longevity (surveys: median 11.45 years, range 6.3–14.3; veterinary hospital records: median 6.5 years, range 3.5–9.1).

### Breed Behavior and Popularity

Pearson's correlations between breed behavioral scores and popularity, volatility, and rates of increase and decrease around popularity peaks are shown in Figure 2. Care is usually required in interpreting the results of multiple statistical tests because of the increased risk of false positives. In the present case, however, only 2 correlations out of 56 are significantly different from zero at the 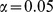, even before any correction for multiple tests. No correlation is significant under the Bonferroni correction, which aims at keeping at 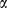 the probability of one or more false positives and would require 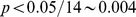 (when applied separately to each family of 14 comparisons between one measure of popularity and one C-BARQ variable). The procedure by Benjamini & Yekutieli [19], which keeps at 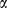 the expected proportion of false positives and is therefore much less conservative, also reveals no significant correlation (adjusted 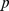 values are reported in the third table columns of Figure 2).

### Breed Longevity and Popularity

Pearson's correlations between popularity and longevity are not significant, with longevity estimated either from surveys (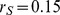, 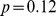, 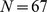) or from veterinary hospital records (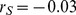, 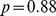, 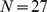).

### Breed Health and Popularity

**Figure 3:**
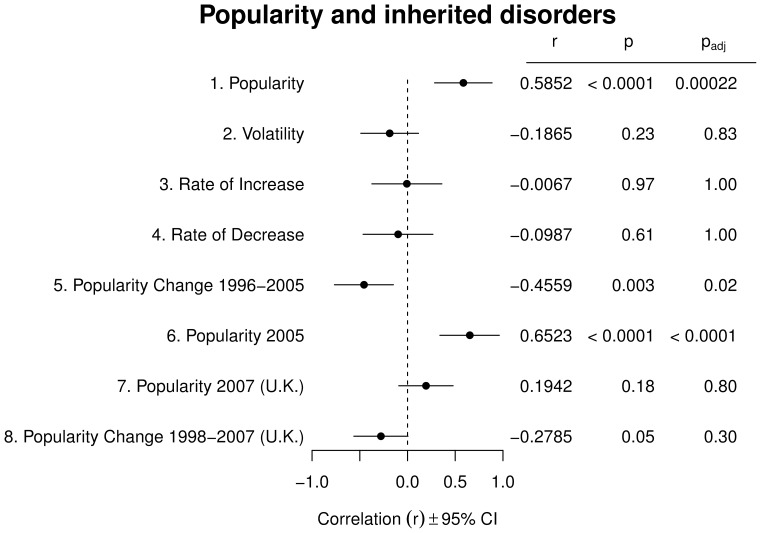
Inherited deseases and breed popularity. Pearson's correlations between number of inherited diseases per breed and indicators of breed popularity in the U.S. and U.K. See legend to Figure 2 for details of the display. Sample sizes for rows 1–8 are, respectively: 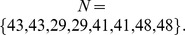

Figure 3 reports correlations between the number of inherited disorders affecting a breed and aspects of breed popularity in the U.S. and the U.K. We find a strong positive correlation between a breed's popularity in the U.S. and the number of inherited disorders from which the breed suffers (row 1), and no correlation between number of disorders and breed volatility or rates of increse and decrease around popularity peaks (rows 2–4). We also find that breeds with more disorders have decreased in popularity in 1996–2005 (row 5), a result that parallels a similar, albeit weaker trend observed in U.K. data (row 7, originally reported in [17]). Altogether, these findings suggest that popular breeds carry a significant health burden, and that in recent years the public may have started to avoid breeds with more health problems. The latter effect is statistically significant, but not large: number of inherited disorders explains only about 10% of the variance in popularity changes in both the U.S. and the U.K. (as estimated from ANOVAs with popularity change as the dependent variable and number of disorders as the independent variable). Indeed, number of disorders was still an excellent predictor of popularity in the U.S. in 2005 (row 6), although it did not strongly correlate with popularity in the U.K. in 2007 (row 8).

## Discussion

We found no indication that breeds with more desirable behavior, longer life, or fewer inherited genetic disorders have been more popular than other breeds. Indeed, inspection of Figure 2 reveals that most of the correlations we report, albeit non significant, run contrary to the hypothesis that breeds with better behavior or temperament have been more popular. For example, we found a negative correlation between popularity and trainability, and a positive correlation between popularity and separation problems, fear of other dogs, and aggression directed toward the owner. Only the negative correlation between popularity and the propensity to chase other animals (a common complaint among dog owners) agrees with the hypothesis that more popular breeds are better behaved. If anything, our results suggest that breeds can become popular despite problematic behavior, rather than because of good behavior.

We found, likewise, that breeds with more inherited disorders have been more popular, rather than less popular, suggesting that health considerations have been secondary in the decision to acquire dogs as well as in dog breeding practices. The disorders accrued by popular breeds are likely to constitute a significant welfare burden to animals: [16] and [17] documented a median of 32.5 inherited disorders per breed, with 25% of breeds suffering from 45 or more disorders. A recent, slight decrease in the popularity of breeds more affected by inherited disorders may indicate that the public is increasingly concerned with the ethical and/or financial implications of this burden (the average cost of owning a medium-sized dog is estimated at USD 8,000, which can increase considerably in the case of illness [20]).

Our results suggest that fashion (social influence) has been more important than function (intrinsic features of the cultural traits at stake) in determining the popularity of dog breeds. The distinction between fashion and function is prominent in cultural evolution studies. Approaches inspired by evolutionary biology [21] have generally stressed the role of social influence (fashion) in individual choices. The core idea is that individuals often decide which cultural variant to adopt according to the social context in which they face the choice. For example, one may adopt a variant relying on information on its frequency (the most studied case being conformism, or copying the majority [22], [23]), or relying on information on individuals that bear the variant (for example, by copying prestigious individual, [24], or by copying individuals of similar age [25]). The possibility that individuals evaluate the characteristics of cultural variants is also acknowledged (called “direct bias” or “content bias”), but it has been studied less because, unless one knows how variants are evaluated, the only conclusion that can be drawn is, trivially, that preferred variants will be chosen more often than non-prefereed ones [21].

Other research traditions emphasize instead the importance of intrinsic features of cultural traits by making specific assumptions about how variants are chosen. According to rational choice theory in economics, for example, individuals evaluate costs and benefits of different variants and make choices that maximises their personal advantage [26]. While this paradigm has been criticised as too rigid to accommodate the quirks of human choices [27], [28], it is clear that individuals can assess (or, in any case, attempt to assess), whether a variant is valuable or not. Cognitive anthropology has also emphasized content-based choice by assuming that certain features of cultural variants make them intrinsically more appealing [29]. Some variants, for example, may elicit strong emotional reactions, such as disgust [30], or be appealing because they convey valued information, such as information about others [31], [32].

Our results contribute to this debate with the strong indication that, in the choice of which dog breed to adopt, context is more important than content. This conclusion appears warranted also in light of recent models of fashions, which have been able to reproduce statistical regularities of fashion cycles based purely on social influence [7]. More work is needed to assess whether our conclusion holds across cultural domains, and to further elucidate the interplay between fashion and function in the choice of cultural variants.
